# Polymeric Materials in Biomedical Engineering: A Bibliometric Mapping

**DOI:** 10.3390/polym17212886

**Published:** 2025-10-29

**Authors:** Cristina Veres, Maria Tănase, Dan-Alexandru Szabo

**Affiliations:** 1 Department of Industrial Engineering and Management, George Emil Palade University of Medicine, Pharmacy, Science, and Technology of Targu Mures, Nicolae Iorga Street 1, 540088 Targu-Mures, Romania; cristina.veres@umfst.ro; 2 Mechanical Engineering Department, Petroleum-Gas University of Ploiesti, 100680 Ploiesti, Romania; 3 Department M2, Faculty of Medicine, George Emil Palade University of Medicine, Pharmacy, Science, and Technology of Targu Mures, Gheorghe Marinescu Street 38, 540139 Targu Mures, Romania; dan-alexandru.szabo@umfst.ro

**Keywords:** polymeric materials, biomedical engineering, tissue engineering, drug delivery, wound healing, hydrogels

## Abstract

This study offers an integrated synthesis of polymeric materials in biomedical engineering, revealing four major and interlinked research domains: tissue engineering and regenerative medicine, drug delivery and nanomedicine, wound healing and antimicrobial applications, and advanced fabrication through 3D/4D printing and bioprinting. Across these areas, hydrogels, biodegradable composites, and stimuli-responsive polymers emerge as the most influential material classes. The analysis highlights substantial progress in extracellular matrix–mimetic scaffolds, smart drug delivery systems with controlled release, multifunctional wound dressings integrating antimicrobial and healing functions, and patient-specific constructs produced via additive manufacturing. Despite these advances, recurring challenges persist in long-term biocompatibility and safety, scalable and reproducible fabrication, and regulatory standardisation. The results point toward a convergence of bioactivity, manufacturability, and clinical translation, with hybrid natural–synthetic systems and personalised polymeric designs defining the next phase of biomedical polymer innovation.

## 1. Introduction

Biomaterials are substances engineered to interact with biological systems for the purpose of evaluating, treating, augmenting, or replacing any tissue, organ, or bodily function [1]. The history of biomaterials can be categorised into generations based on their functional objectives. The earliest, first-generation materials were designed to be bio-inert, simply coexisting with the host without eliciting a toxic response [2]. Over time, the field evolved, leading to a third generation of materials that are not merely inert but are designed to actively promote or inhibit specific biological activities, such as cell adhesion and tissue regeneration [2,3]. This shift has been catalysed by the increasing demand for sophisticated medical interventions that not only repair but also restore physiological function.

Within this evolving landscape, polymeric materials have emerged as the basis of modern biomedical engineering. Their ascendancy is a result of their distinct advantages over traditional materials, such as metals and ceramics. Polymers offer remarkable versatility, with the ability to be synthesised with customised chemical and physical properties that suit a wide range of applications [1]. They are also lightweight and possess a lower coefficient of thermal expansion than metals, which can be particularly beneficial for load-bearing applications, as it minimises stress on surrounding tissues [4]. The ability to specifically modify their properties for different parts of the body has made them a centre of innovation and commercial development [5].

The foundational material science of polymeric biomaterials is rooted in a fundamental choice between two distinct classes of materials: natural and synthetic polymers. This choice presents an essential trade-off between intrinsic biological compatibility and mechanical or chemical properties.

Natural polymers are derived from living organisms, including plants, animals, and microorganisms [6,7]. Their primary advantage lies in their inherent biological compatibility and their structural similarity to the native extracellular matrix (ECM) of the human body [7,8]. This biomimicry enables them to be recognised by the body, thereby minimising the risk of chronic inflammatory reactions, toxicity, or immunological rejection [7,8]. Examples of natural polymers widely used in biomedical applications include proteins such as collagen, fibrin, and silk, as well as polysaccharides like chitosan and hyaluronic acid [7]. Despite these benefits, they are mechanically inferior to synthetic alternatives and are susceptible to batch-to-batch variability [7]. Furthermore, since they are derived from biological sources, they carry a potential risk of disease transmission or immunogenicity [1].

In contrast, synthetic polymers are artificially created in laboratories, offering a high degree of control over their properties [7,9]. They can be produced under controlled conditions with predictable and reproducible characteristics, including mechanical strength, degradation rate, and chemical composition. This tunability allows researchers to engineer materials with properties tailored for specific functions, such as the tensile strength required for a suture or the elasticity needed for a cardiovascular graft [1]. Widely studied examples include polylactic acid (PLA), poly(glycolic acid) (PGA), poly(caprolactone) (PCL), and their copolymer PLGA [10]. The primary disadvantage of synthetic polymers is their potential for poor biocompatibility and a general lack of cell adhesion sites, which often necessitates chemical modifications or surface treatments to enhance their integration with biological tissue [7].

Table 1 presents a comparison between natural and synthetic polymers used for biomedical applications.

The distinct advantages and disadvantages of natural and synthetic polymers have created a clear and persistent dilemma in the field. Natural polymers are inherently well-tolerated by the body but lack the robust and reproducible mechanical properties required for many applications. Synthetic polymers offer unparalleled mechanical and chemical control, but they also pose risks of adverse biological reactions. The logical progression of research has been to bridge this gap by developing hybrid systems that combine both polymer types [15,16]. This approach is not a simple mixture but a sophisticated engineering strategy that leverages the mechanical strength and reproducibility of synthetic polymers while benefiting from the superior bioactivity and biocompatibility of natural ones [17,18]. The creation of these synergistic, hybrid materials represents a fundamental shift away from the earlier paradigm of single-material biomaterials. The most promising future materials are therefore not pure but are intelligently designed composites that actively integrate the strengths of both classes to meet the complex demands of biomedical applications.

The economic significance of this field is substantial, reflecting its rapid growth. In 2013, the global market for implantable biomaterials was valued at nearly 75.1 billion USD and was projected to reach 109.5 billion USD by 2019, growing at a compound annual growth rate (CAGR) of 6.7% [1,19]. The polymeric biomaterials sector was identified as a key driver of this expansion, with a projected CAGR of 22.1% [1,19]. More recent data reinforce this trend, showing an astonishing growth surge with the polymer biomaterial market escalating from 79.06 billion USD in 2024 to 94.98 billion USD in 2025, a CAGR of 20.1% [20]. Projections indicate that this market will continue its robust expansion, potentially reaching 169.88 billion USD by 2029 [20]. This growth is geographically concentrated, with North America and the Asia-Pacific region anticipated to dominate the market, reflecting a high concentration of research and healthcare investment [21].

Given the rapid evolution and growing complexity of this field, a comprehensive synthesis of the existing literature is warranted. This paper presents a review of highly cited review articles to provide a high-level, authoritative synthesis of the current state of knowledge. The aim is to identify key findings and knowledge gaps and to outline future directions in the role of polymers in biomedical applications, thereby providing a foundational reference for researchers and industry professionals.

## 2. Applications and Innovations: A New Frontier for Healthcare

Polymeric materials are not merely substitutes for other materials; they are enablers of new medical technologies. Their versatility has led to transformative applications across three primary domains: tissue engineering, controlled drug delivery, and advanced medical devices.

Table 2 presents some examples of polymers and their specific medical applications.

### 2.1. Tissue Engineering and Regenerative Medicine

The core function of polymers in tissue engineering (TE) is to design and construct scaffolds that provide a three-dimensional environment for tissue regeneration [40,41,42,43]. These scaffolds are bio-mimetic structures that emulate the natural ECM, offering mechanical, spatial, and biological cues that guide cellular responses, including adhesion, proliferation, and differentiation [44,45]. A key design objective is to ensure that the scaffold degrades at a rate commensurate with new tissue formation, allowing the new tissue to gradually assume mechanical load as the polymer recedes [46,47,48,49].

The field has advanced from simple scaffolds to complex, patient-specific structures. For example, in spinal cord regeneration, scaffolds loaded with neural progenitor cells (NPCs) have been bioprinted to accelerate axon regeneration and improve motor function in animal models [50,51]. Similarly, for cartilage repair, 3D printed scaffolds can be designed with complex, irregular shapes that mimic the natural geometry of tissue, allowing for the reconstruction of structures such as auricular or tracheal cartilage [50,52,53]. Bone regeneration has also seen significant advances with the development of composites. The addition of hydroxyapatite (Hap) to polymeric scaffolds has been shown to improve bioactivity and promote the growth of a mineral layer that closely mimics natural bone [54,55,56,57,58].

### 2.2. Controlled Drug Delivery Systems (DDS)

Polymers are indispensable for controlled drug delivery, serving as a primary means to manage medication release, protect drugs from physiological degradation, and enhance patient compliance by reducing the frequency of dosing [59,60,61,62].

The stealth effect enhances nanomaterial pharmacokinetics by improving blood circulation and tissue targeting, yet most systems still show rapid clearance after administration—a phenomenon termed the pseudo-stealth effect. Achieving true stealth behaviour requires a holistic surface design, where overall structure, charge, and hydrophobicity are optimised rather than relying solely on single polymer coatings such as PEGylation [63]. A significant innovation within this area is the development of “smart” or stimuli-responsive polymers [64]. These materials are designed to change their physical or chemical properties in response to specific environmental triggers [61]. These triggers can be internal, such as the acidic pH of a tumour microenvironment, the presence of specific enzymes, or changes in glucose levels [65,66,67,68]. Alternatively, they can be external, like light, ultrasound, or magnetic fields [65,66,67,68,69]. This responsiveness enables precise, site-specific drug delivery, thereby maximising therapeutic efficacy while minimising off-target effects and systemic toxicity. This level of control is fundamental to the advancement of personalised medicine, where treatments can be tailored to an individual’s unique physiological or pathological state [70].

### 2.3. Innovations in Medical Devices and Implants

Polymers are used in a vast spectrum of medical devices, from simple, single-use items to complex, long-term implants [71,72,73]. Their utility extends to every aspect of healthcare, from surgical tools and wound dressings to advanced prosthetic devices. For instance, ultrahigh-molecular-weight polyethylene (UHMWPE) is the material of choice for load-bearing components in hip and knee replacements [74]. Polyurethanes (PU) are prized for their high biocompatibility and hemocompatibility, making them ideal for applications such as artificial hearts [75,76,77,78,79,80], catheters [81,82,83], and wound dressings [84,85,86]. Poly(ether ether ketone) (PEEK) is increasingly employed in long-term orthopaedic implants [28,87,88,89].

The convergence of two major trends—customisation and adaptability—is shaping the next generation of these devices. Additive manufacturing, specifically 3D printing and bioprinting, enables the creation of patient-specific implants and scaffolds derived from medical imaging data [90,91,92,93,94,95,96,97]. Simultaneously, the development of smart polymers allows for materials that are adaptive to a patient’s unique physiological state, such as fluctuating glucose levels or the acidic pH of a cancerous tumour. This convergence of customisation and adaptability is poised to usher in a new era of dynamic, responsive systems that are fully integrated into an individual’s biology, marking an actual realisation of the promise of personalised medicine.

## 3. Materials and Methods

This study was designed as a bibliometric mapping combined with a structured synthesis of highly cited review papers on polymeric materials in biomedical engineering. The rationale for focusing on review articles was to consolidate established evidence and consensus knowledge, providing a reliable foundation for identifying research trends, methodological advances, and persisting challenges. While this design ensures a comprehensive and validated overview of the field, it may also introduce a degree of bias toward mature or well-explored topics, potentially underrepresenting emerging or niche research areas not yet reflected in major reviews. Acknowledging this trade-off, the analysis emphasised the interpretative depth of the selected reviews rather than the sheer volume of data, aiming to distil the most significant findings shaping the field.

First, a bibliometric analysis was conducted using the Web of Science Core Collection, selected for its rigorous indexing and broad coverage of biomedical research.

Second, a synthesis of the most cited review articles (≥100 citations) was performed, to highlight the dominant themes, methodological advances, and persistent challenges reported across the literature. By analysing these highly influential works, the study aimed to identify knowledge gaps and outline future research trajectories.

The search was conducted using the query: (“polymeric materials” OR “biomedical polymers” OR “polymeric biomaterials”) AND (“biomedical” OR “medical” OR “healthcare” OR “tissue engineering” OR “drug delivery” OR “implant”) AND (“Review” OR “Systematic Review”). This search returned 1095 records.

The results were refined by restricting the document type to reviews, which yielded 844 articles. The search was then restricted to the period 2016–2025, which reduced our dataset to 628 articles. No duplicates were found. Then titles and abstracts were screened to confirm alignment with the research objective. Finally, the full texts of the remaining articles were examined, with inclusion limited to those reviews that addressed biomedical applications of polymers.

By manual scanning, 39 articles were excluded because their field of study did not align with the scope of this review, which focuses on biomedical applications of polymeric materials.

More specifically, the excluded articles belonged to the following broader domains:

- Fundamental and synthetic chemistry (15 articles);
- Energy, environment, and sustainability (7 articles);
- Plant- and marine-derived biopolymers (3 articles);
- Nanomaterials and coatings for non-medical applications (5 articles);
- Engineering and additive manufacturing outside biomedical focus (4 articles) *;
- Electronics and supramolecular nanostructures (3 articles);
- Miscellaneous non-biomedical topics (2 articles): where the emphasis was on interdisciplinary areas without substantive medical or healthcare relevance.

* Reviews were excluded if they addressed polymeric materials used exclusively for non-medical purposes, such as industrial coatings or packaging. In addition, reviews focused on engineering and additive manufacturing were included only when these technologies were applied to biomedical contexts. For instance, studies centred on mechanical optimisation of 3D printing parameters for structural polymers, without reference to biocompatibility or medical devices, were excluded.

**Figure 1:**
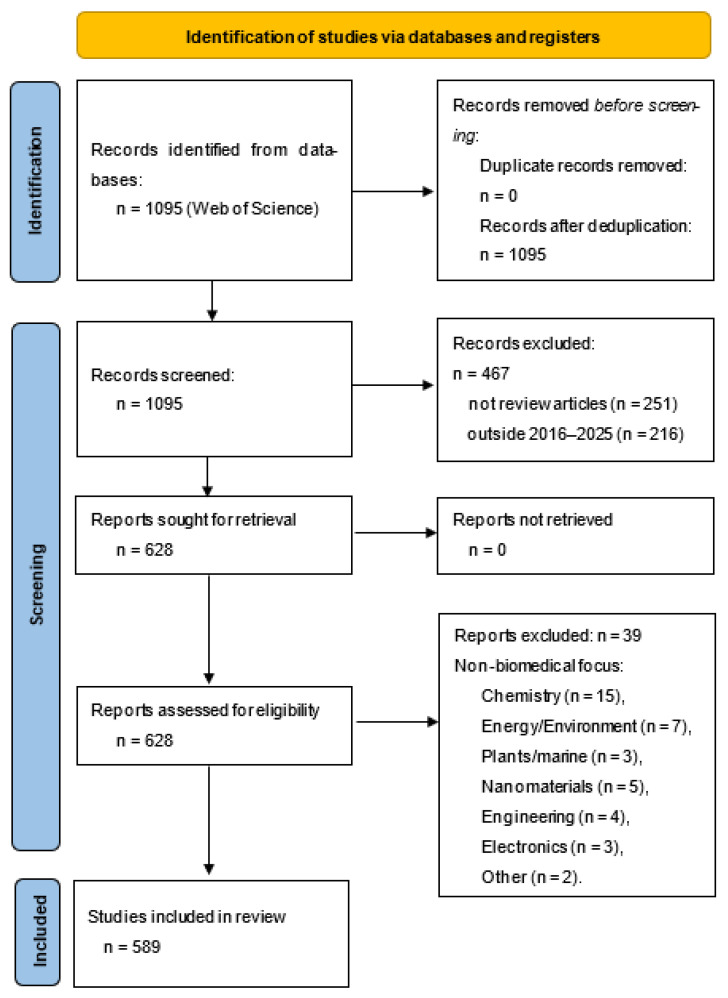
PRISMA flow for bibliometric selection.

Ultimately, the research encompassed 589 review papers. The study selection process is summarised in the PRISMA flow diagram (Figure 1), which details the identification, screening, eligibility, and inclusion stages of the review (Table S1 in the Supplementary).

The bibliometric analysis was performed using VOSviewer (version 1.6.20), a software tool designed to map and visualise scientific literature. Co-occurrence networks of keywords, bibliographic coupling among sources, and international co-authorship maps were generated. Both overlay and density visualisations were used to illustrate temporal and structural patterns.

**Figure 2:**
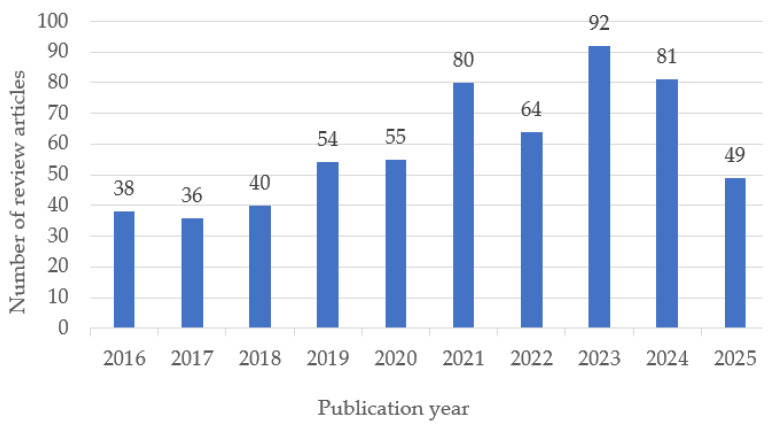
Annual distribution of review articles on biomedical polymers included in the study (2016–2025). Note: Data for 2025 are partial, as the year is ongoing; therefore, the apparent decline reflects incomplete indexing rather than a decrease in publication activity.

The temporal distribution of the selected review articles shows a steady growth in publications on biomedical polymers between 2016 and 2025, with a peak in 2023 (Figure 2). This trend reflects the increasing scientific interest and diversification of research topics in recent years.

## 4. Bibliometric Analysis of Polymeric Materials in Biomedical Engineering

**Figure 3:**
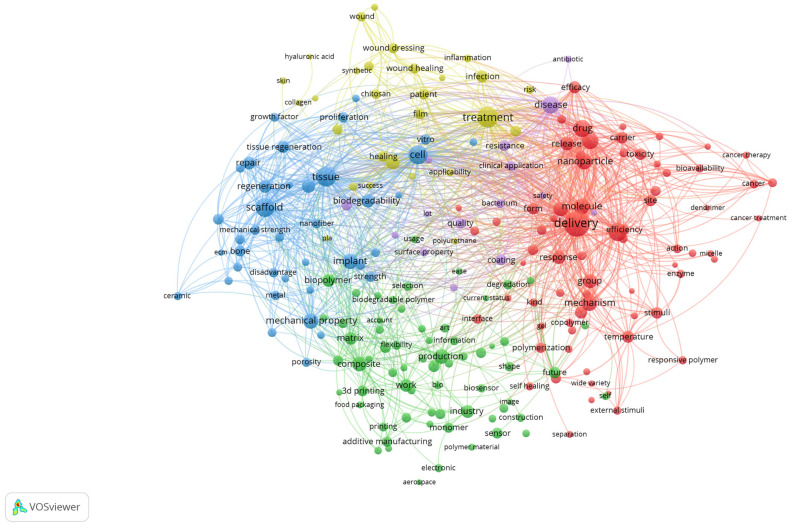
Keyword co-occurrence network of biomedical polymer research, based on text data.

A co-occurrence analysis of terms derived from the titles and abstracts of the included review articles was performed using VOSviewer with binary counting, which considers the presence of a term within a document regardless of its frequency of occurrence (Figure 3). The generated map visualises the structural relationships among recurrent concepts and reveals the main thematic directions in biomedical polymer research.

The red cluster, situated on the right side of the map, is centred around terms such as “drug,” “delivery,” “nanoparticle,” “cancer,” and “toxicity”. This cluster emphasises the extensive exploration of polymeric systems as nanocarriers, particularly in oncology, where controlled release, targeting strategies, and responsiveness to biological stimuli represent dominant research trends. The strong linkages within this cluster highlight the convergence of drug delivery science, cancer therapy, and polymer chemistry.

The blue cluster, positioned on the left, brings together terms such as scaffold, tissue, regeneration, bone, repair, implant and cell. This cluster underscores the importance of tissue engineering and regenerative medicine, where polymeric scaffolds, both natural and synthetic, are evaluated for their ability to replicate extracellular matrix properties and promote cell proliferation, differentiation, and tissue repair. The interconnections with terms like implant and biodegradability indicate a focus on clinical translation.

The green cluster, located in the lower part of the map, comprises terms including production, composite, 3D printing, additive manufacturing, and industry. This group highlights the importance of material science and engineering aspects of biomedical polymers, where optimising mechanical strength, porosity, and processing techniques is crucial for ensuring functionality in clinical devices. The inclusion of additive manufacturing terms indicates the growing importance of advanced fabrication methods in customising polymer-based biomaterials.

The yellow cluster, located in the upper part of the visualisation, is characterised by terms such as healing, treatment, infection, film, wound dressing, and patient. This cluster captures the translation of polymeric materials into clinical solutions for wound management and infection control. The presence of both synthetic and natural polymers within this cluster suggests ongoing efforts to strike a balance between cost-effectiveness, biodegradability, and therapeutic performance in real-world healthcare applications.

Taken together, the map demonstrates that the field of biomedical polymers is structured along two complementary axes: (i) the development of advanced materials and fabrication strategies, and (ii) their translation into biomedical applications ranging from targeted drug delivery and cancer therapy to tissue regeneration and wound healing. The overlaps among clusters underscore the interdisciplinary nature of the field, where chemistry, engineering, and clinical sciences converge to shape emerging research frontiers.

**Figure 4:**
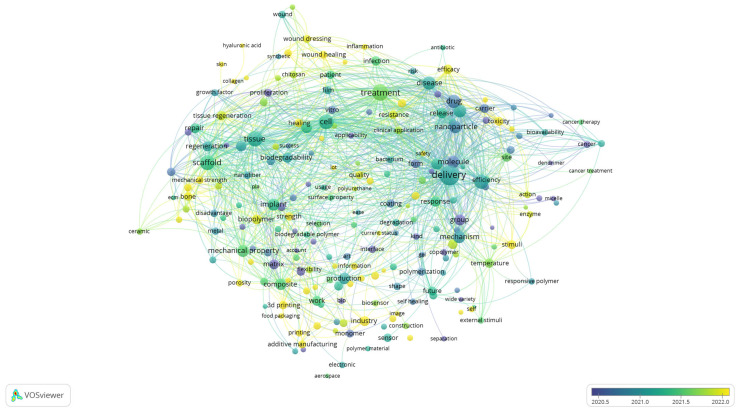
Overlay visualisation of keyword co-occurrence, showing temporal evolution of research themes, based on text data.

While the cluster visualisation highlights the structural composition of the research field by grouping related terms into thematic clusters, the overlay visualisation provides a complementary perspective by incorporating the temporal dimension. By colouring terms according to their average year of occurrence, Figure 4 enables the tracing of how the focus of biomedical polymer research has shifted over time. In this way, the analysis identifies the dominant thematic areas and reveals which topics are well-established and which ones are emerging more recently.

Although the scale displayed in the figure emphasises the most active period from 2020 to 2022, the underlying dataset encompasses the entire decade. Earlier contributions (shown in blue and green) are associated with terms such as “molecule, “drug,” “in vitro,” “tissue,” and “cancer”, reflecting the foundational focus on drug design, experimental validation, and the early application of polymers in biomedical and oncological contexts. Terms positioned in the central spectrum (greenish tones), including delivery, nanoparticle, biodegradability, and treatment, represent themes that have remained consistently relevant across the years, particularly in drug delivery systems, nanomedicine, and biocompatibility assessment. More recent developments (yellow shades) highlight emerging directions such as wound healing, 3D printing, biopolymer, industry, and innovation, pointing to a growing emphasis on translational research, advanced manufacturing strategies, and the integration of polymer science into clinical and industrial applications.

This temporal distribution suggests that the field has progressed from a predominantly fundamental orientation, focused on molecular design, in vitro validation, and early biomedical applications, toward more application-driven and translational research.

Based on the bibliographical data, additional visualisations were generated to provide deeper insights into the structural composition and thematic development of the field. A co-occurrence analysis of author keywords was performed in VOSviewer using complete counting with a resolution parameter set at 0.70.

**Figure 5:**
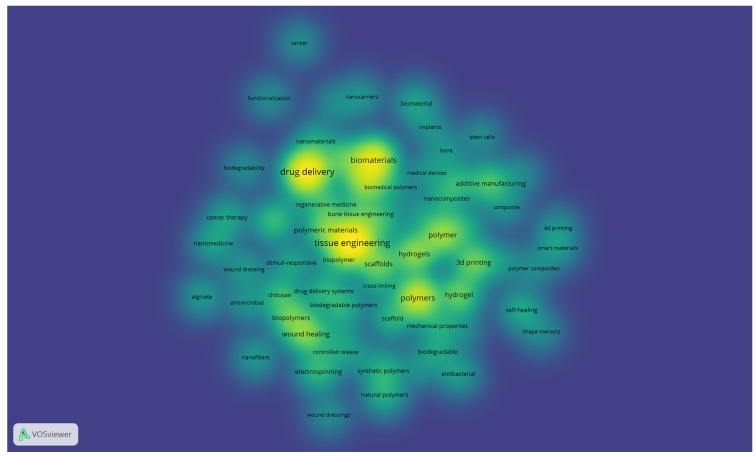
Density visualisation of author keywords in biomedical polymer research (VOSviewer, resolution = 0.70, full counting).

The resulting density visualisation (Figure 5) highlights the most frequently used terms, with brighter areas indicating higher occurrence and stronger interconnections. Central concepts, such as tissue engineering, drug delivery, biomaterials, and polymers, emerge as dominant research fronts, surrounded by related themes including hydrogels, 3D printing, wound healing, and nanomaterials. This distribution reflects the interplay between foundational topics in polymer science and their translation into clinical and technological applications, offering a representation of the knowledge structure in biomedical polymer research.

**Figure 6:**
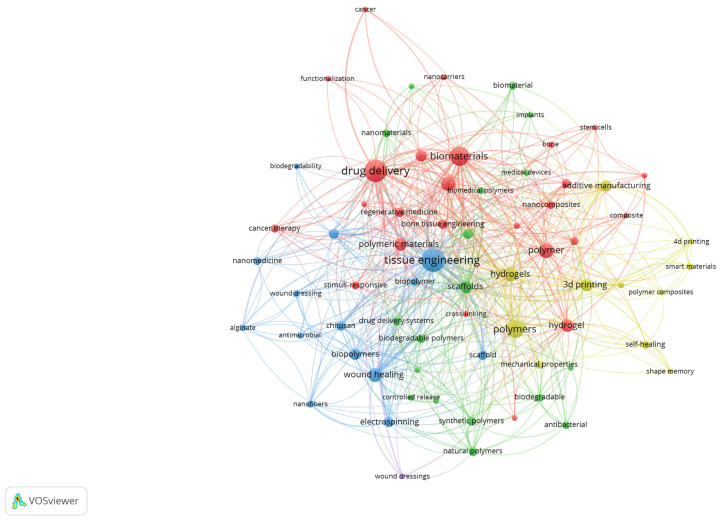
Network visualisation of author keywords in biomedical polymer research.

To better illustrate the structural relationships among the most relevant terms, a network visualisation of author keywords was also generated. Figure 6 presents the co-occurrence clusters, where nodes represent keywords and their size reflects the frequency, while the links indicate the strength of co-occurrence. The colour-coded clusters highlight distinct thematic areas, such as tissue engineering, drug delivery, biomaterials, and additive manufacturing, emphasising the interdisciplinary connections that characterise biomedical polymer research.

The red cluster is centred on drug delivery and biomaterials, emphasising the extensive exploration of polymer-based carriers for controlled release, cancer therapy, and the design of biocompatible systems. The blue cluster groups terms such as tissue engineering, scaffold, wound healing, and nanofibers, reflecting the intense focus on regenerative medicine and the development of polymeric scaffolds that mimic extracellular matrices. The green cluster includes keywords related to biomedical polymers, biodegradability, nanomaterials, and natural polymers, indicating the increasing interest in sustainable, bio-inspired materials with advanced functionalities. Finally, the yellow cluster brings together 3D printing, additive manufacturing, smart materials, and hydrogels, highlighting the emerging role of additive manufacturing and advanced fabrication strategies in tailoring polymer properties for clinical applications. Together, these clusters illustrate both the consolidation of established research directions and the diversification toward novel, application-driven topics.

**Figure 7:**
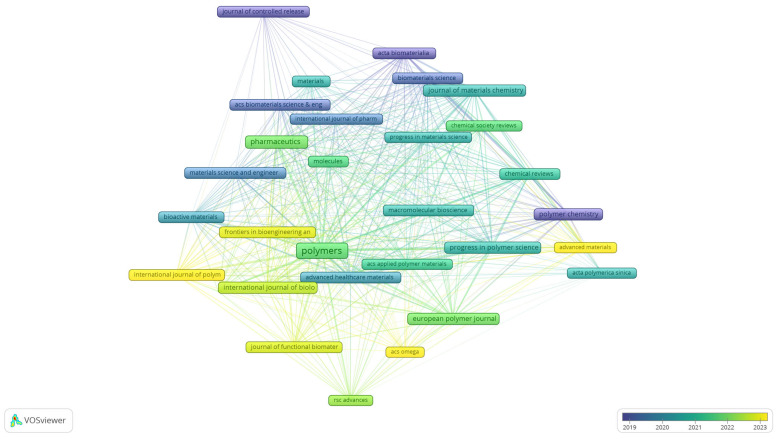
Overlay visualisation of bibliographic coupling among sources in biomedical polymer research.

To complement the keyword analyses, the bibliographical dataset was further examined through bibliographic coupling of sources, providing an overview of the journals that shape the field of biomedical polymer research. Figure 7 presents the overlay visualisation of this network, where node size reflects the number of documents, the thickness of the links indicates the strength of shared references, and the colour scale corresponds to the average year of publication.

The map indicates that long-established journals, such as the Journal of Controlled Release, Acta Biomaterialia, and Biomaterials Science (represented by blue to green tones), have consistently provided a foundation for the field, with a focus on drug delivery, biomaterials development, and materials chemistry. More recent contributions are concentrated around open-access and interdisciplinary outlets, including Polymers, Pharmaceutics, and Chemical Reviews, as well as the Journal of Materials Chemistry (green to yellow), reflecting the expansion of the field toward broader dissemination and increased accessibility. The emerging presence of titles such as RSC Advances, ACS Omega, and Advanced Materials indicates a diversification of publication venues in recent years. This distribution highlights the coexistence of high-impact, specialised journals with newer, more inclusive platforms, underscoring both continuity and renewal in the dissemination of biomedical polymer research.

**Figure 8:**
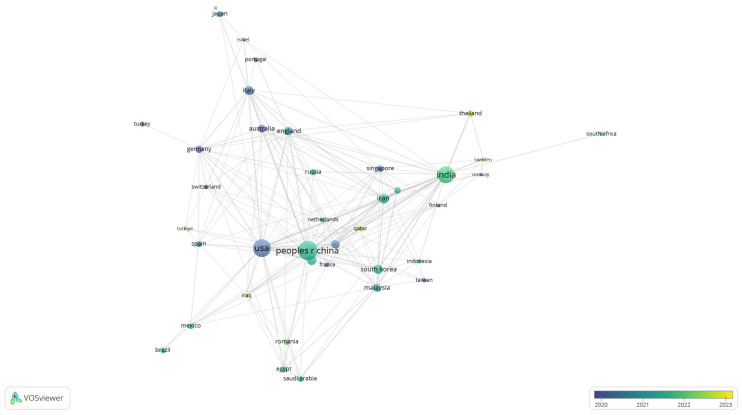
Overlay visualisation of international co-authorship in biomedical polymer research.

To further explore patterns of collaboration, a co-authorship analysis was conducted at the country level. Figure 8 displays the network of international cooperation in biomedical polymer research, where node size corresponds to the number of documents and the colour scale reflects the average year of publication.

The visualisation highlights the central role of leading contributors, including the United States, China, and India, which not only dominate in terms of publication volume but also establish strong collaborative links with European and Asian partners. Countries like Germany, England, and Australia appear as important bridges in the network.

Established contributors with earlier average years include the USA, China, Germany, England, Italy, Japan and the Netherlands (blue to green), reflecting long-standing output and dense collaboration ties. By contrast, more recent and growing contributors—most visibly India, Sweden, Iraq, Turkey, Thailand and Romania (yellow hues)—signal increased engagement during 2022–2023.

Overall, the bibliometric mapping highlights both the consolidation of established domains—such as tissue engineering, drug delivery, and wound healing—and the emergence of newer directions, including additive manufacturing, smart polymers, and bio-inspired biomaterials. The temporal overlay visualisations confirmed a shift from fundamental, chemistry-driven studies to more application-oriented and translational research, while the density and network maps emphasised the centrality of hydrogels, scaffolds, and nanomaterials in the current discourse. The coupling analysis of journals demonstrated a balance between long-established, high-impact outlets and newer open-access platforms. In contrast, the co-authorship networks revealed the increasing globalisation of the field, with substantial contributions from the United States, China, India, and an expanding presence of European partners. Taken together, these results provide a structured framework that guided the subsequent qualitative synthesis of the most highly cited review articles, allowing for a deeper understanding of how research fronts are shaped and where future opportunities may lie.

## 5. Analysis of the Most Cited Review Articles

To complement the bibliometric mapping, a qualitative analysis was conducted on the most cited review articles within the dataset. Articles with over 100 citations were selected as representative of the field’s most influential contributions. This approach allowed for the identification of dominant themes, methodological advances, and knowledge gaps reported across highly visible works, providing additional depth to the quantitative mapping results.

Out of the 589 review papers, 90 reached this threshold of more than 100 citations. Table 3 presents these articles, including information on authorship, research area, and primary focus, together with a concise description of their key findings and the number of times they have been cited.

The synthesis of the 90 most cited review articles highlights the diversity and maturity of research on polymeric biomaterials. While the individual studies cover a wide spectrum of applications, several dominant thematic lines emerge across the literature. These include tissue engineering and regenerative medicine, controlled drug delivery and nanomedicine, wound healing and antimicrobial strategies, as well as the adoption of advanced fabrication methods such as 3D printing and bioprinting. At the same time, recurring methodological concerns—such as reproducibility, scalability, and long-term clinical validation—are repeatedly emphasised, highlighting gaps that remain insufficiently addressed despite the field’s rapid expansion.

Based on the distribution of the most cited reviews, the literature can be broadly grouped into four major thematic domains: (i) tissue engineering and regenerative medicine, (ii) drug delivery and nanomedicine, (iii) wound healing and antimicrobial applications, and (iv) advanced fabrication strategies such as 3D printing and bioprinting. Each of these domains reflects both the consolidation of established research fronts and the emergence of novel directions that shape the field of polymeric biomaterials.

(i) Tissue engineering and regenerative medicine

A substantial proportion of the highly cited reviews focus on polymer-based scaffolds designed to mimic the extracellular matrix and support cell growth, differentiation, and tissue repair. Hydrogels, composite materials, and bio-inspired scaffolds dominate this category, reflecting the centrality of tissue regeneration in biomedical polymer research. Reviews such as those by Nikolova & Chavali [99], Reddy et al. [9], and Islam et al. [103] emphasise advances in scaffold fabrication, electrospinning techniques, and the incorporation of bioactive molecules to enhance regeneration. Additional contributions, including those by Bai et al. [104] and Boni et al. [110], discuss hydrogels for bone and neural tissue applications. Meanwhile, Kennedy et al. [131] and Abbasian et al. [134] highlight cell–matrix interactions and the potential of natural macromolecules in scaffold design. Despite these advances, persistent challenges remain in achieving controlled degradation, mechanical stability, and full integration into host tissue, underlining tissue engineering as a long-standing but still evolving frontier.

(ii) Drug delivery and nanomedicine

Another major cluster of reviews centres on polymeric nanoparticles and stimuli-responsive polymers for targeted drug delivery. Begines et al. [100], Karimi et al. [108], and Tang et al. [112] highlight how smart polymer systems enable controlled release profiles, responsiveness to biological stimuli, and enhanced therapeutic efficacy in oncology and chronic diseases. Other reviews, such as Bernard et al. [119], Bagheri et al. [118], and Khan et al. [129], extend the discussion to biocompatibility, lanthanide-doped systems, and brain-targeted nanoformulations. Similarly, Essa et al. [130], Elmowafy et al. [153], and Shrimal et al. [170] analyse PLGA-based carriers, bioactive natural agents, and novel nanoparticle preparation methods. Across these studies, concerns regarding systemic toxicity, large-scale production, and regulatory hurdles are consistently reported, suggesting that while laboratory-scale innovations are promising, their translation into standardised clinical applications remains incomplete.

(iii) Wound healing and antimicrobial applications

Hydrogels and polymer-based coatings for wound care and infection control constitute a third recurring theme. Reviews by Zhang et al. [98], Varaprasad et al. [102], and Sharma et al. [136] illustrate the potential of catechol-functionalized hydrogels, chitosan-based composites, and nitric oxide-releasing polymers to accelerate healing and provide antimicrobial protection. Complementary perspectives are provided by Cho et al. [109], Sánchez-Cid et al. [123], and Mushtaq et al. [124], who report on adhesive hydrogels, multifunctional gelatin-based systems, and hybrid composites. More recent works, including those by Cai et al. [154], Kaniuk & Stachewicz [155], and Gnanasekar [160], suggest the use of electrospun fibres, PHBV-based biodegradable scaffolds, and engineered polymers for the photodynamic inactivation of pathogens. Despite encouraging laboratory results, unresolved issues related to cost-effectiveness, long-term safety, and comparative clinical performance continue to limit translation into routine healthcare.

(iv) 3D printing, bioprinting, and advanced fabrication

Several of the most cited reviews—such as those by González-Henríquez et al. [106], Culmone et al. [113], and Nouri et al. [144]—position additive manufacturing as a transformative trend in the field. Patient-specific implants, complex scaffolds, and 4D-printed smart polymers are repeatedly emphasised as enabling technologies for personalised medicine. Reviews by Alghamdi [115], Arif et al. [150], and Arif et al. [164] further detail the role of sustainable biomaterials, multifunctional drug-loaded constructs, and the prospects of 4D bioprinting. More specialised perspectives, such as those of Chen et al. [145] on oncology-related applications and Marco-Dufort & Tibbitt [126] on mouldable hydrogels, underscore how advanced manufacturing techniques intersect with biomedical requirements. Across these studies, reproducibility, material standardisation, and regulatory acceptance are consistently identified as barriers, despite the transformative potential of additive manufacturing in bridging the gap between polymer science and clinical practice.

The four major thematic domains align one-to-one with what the VOS maps already show. In Figure 3 text, the blue cluster groups scaffold, tissue, regeneration, bone, repair, implant and cell, which supports the tissue-engineering strand; the red cluster concentrates drug, delivery, nanoparticle, cancer and toxicity, which anchors drug delivery and nanomedicine; the yellow cluster highlights healing, treatment, infection, wound dressing and patient, which maps to wound healing and antimicrobial applications; and the green cluster contains production, composite, 3D printing and additive manufacturing, which substantiates the advanced-fabrication strand. The keyword density and network visualisations reinforce this structure by placing tissue engineering, drug delivery, hydrogels, 3D printing, wound healing and nanomaterials among the most central and tightly connected terms.

By systematically analysing the most influential reviews, we developed an overview of the progress, challenges, and future directions in polymeric biomaterials, complementing the bibliometric mapping and fulfilling the dual objective of mapping and synthesis, as presented in Table 4.

Beyond the descriptive categorization, the synthesis of the 90 most-cited reviews reveals an evolutionary pattern across the decade analysed. Early reviews (2016–2018) focused predominantly on biocompatibility, degradation kinetics, and proof-of-concept demonstrations, reflecting the field’s preclinical orientation. Mid-period studies (2019–2021) progressively shifted toward hybrid and stimuli-responsive systems, where scalability and reproducibility emerged as dominant concerns. The most recent reviews (2022–2024) highlight translational priorities, including clinical validation, manufacturing standardisation, and cost-effectiveness—indicating a maturing field moving from experimental innovation toward clinical implementation.

Interestingly, while there is broad consensus on the importance of hybrid natural–synthetic systems for improving mechanical and biological performance, divergences persist regarding regulatory feasibility and long-term safety standards, especially in smart and degradable polymer systems.

When integrated with the bibliometric overlay map (Figure 4), these qualitative patterns align with the temporal emergence of terms such as wound healing, 3D printing, biopolymer, and innovation (highlighted in yellow), corresponding to the “Future Directions” cluster in Table 4. This convergence underscores that the topics now gaining bibliometric visibility—such as additive manufacturing, responsive hydrogels, and patient-specific biopolymers—are the very directions identified qualitatively as defining the next decade of research.

Taken together, the evidence summarised in Table 4 integrates the quantitative mapping into a coherent narrative of the field. The pattern suggests a gradual shift from materials discovery to application-driven, clinically oriented engineering, while highlighting persistent bottlenecks at the interfaces of biology, manufacturing, and regulation. These insights frame the implications developed next, namely the need for design-for-clinic criteria, harmonised evaluation and reporting practices, and credible pathways to scale up adoption across healthcare settings, which we distil in the Conclusions.

## 6. Conclusions

This article combined bibliometric mapping with a review of the most cited articles to provide a consolidated perspective on the role of polymeric materials in biomedical engineering. The bibliometric analysis demonstrated the rapid expansion and diversification of the field between 2016 and 2025, with clusters converging around tissue engineering, drug delivery, smart polymers, wound healing, and additive manufacturing. International collaboration maps also revealed the increasing global scope of this research, with the United States, China, and India as dominant contributors, complemented by emerging involvement from European and developing countries.

By integrating bibliometric mapping with a structured synthesis of reviewed literature, this study offers a comprehensive reference for researchers, clinicians, and industry stakeholders. The findings underline the need for interdisciplinary strategies that bridge material science, engineering, and clinical practice.

This study has several limitations that should be acknowledged. First, the search was conducted exclusively in the Web of Science Core Collection, which may have excluded relevant reviews indexed in other databases such as Scopus or PubMed. Second, although the inclusion criteria were restricted to the years 2016–2025, some earlier influential reviews might not have been captured. Finally, the bibliometric mapping reflects only the structural and temporal patterns of the selected dataset; therefore, the interpretation of thematic trends should be understood as indicative rather than exhaustive.

The combined use of bibliometric mapping and structured synthesis reveals a clear transition from materials discovery toward application-driven, clinically oriented engineering. Four interlinked research fronts—tissue regeneration, polymer-based drug delivery, wound management, and advanced fabrication—have converged, demonstrating growing translational momentum. Synthesising insights from the 90 most-cited reviews, several recurring solutions emerge: aligning polymer degradation kinetics with tissue healing rates, ensuring sterilisation and imaging compatibility, harmonising validation protocols, and integrating early health-economic assessment. Industrial scalability remains challenged by variability in polymer synthesis, reproducibility of additive-manufactured structures, and the absence of unified regulatory frameworks for hybrid materials.

In summary, the findings suggest that future progress depends on embedding “design-for-clinic” principles within polymer development and strengthening the interface between material innovation, validation, and manufacturing. These integrative pathways provide a concise roadmap for advancing polymeric biomaterials from laboratory innovation to clinical and industrial translation.

## Figures and Tables

**Table 1:** Comparison between natural and synthetic polymers used for biomedical applications.

Property	Natural Polymers	Synthetic Polymers
Source	Living organisms (plants, animals, microorganisms)	Laboratory synthesis (petroleum oil monomers)
Biocompatibility	Inherently excellent, mimics native extracellular matrix ECM	Can be a challenge, may lack cell adhesion sites [7].
Mechanical Strength	Generally inferior and variable	Tuneable, superior strength and durability
Reproducibility	Batch-to-batch variation is common	Consistent and predictable properties
Immunogenicity Risk	Can cause an immune response or disease transmission	Lower risk of immunogenicity/infection
Biodegradability	Controlled enzyme degradation [11]	Degradation rate can be precisely controlled
Examples	Collagen, fibrin, chitosan, alginate, silk, hyaluronic acid [7,11]	PLA, PGA, PLGA, PCL, Polyethylene glycol (PEG), polyurethane (PU) [10,11]
Main advantages	Offer lower toxicity, causing less chronic inflammation or immunological reactions than synthetic polymers, can undergo chemical modifications, are potentially biodegradable and biocompatible, and are cost-effective and readily available for biomedical applications [12]	They are more diverse and versatile for biomedical applications, allowing custom designs and controlled chemical modifications, with tailorable mechanical properties and degradation kinetics, often lower cost than biological scaffolds, scalable production, long storage times, and physical, chemical, and mechanical properties comparable to biological tissues [13,14]
Main disadvantages	These polymers degrade before melting, are difficult to process due to their complex structure, and may transmit diseases from their natural sources [8]	They can trigger an immune response or toxicity when combined with specific polymers that the host tissue cannot integrate. Also, they lack cell adhesion sites and require chemical modifications to improve cell attachment

**Table 2:** Specific medical applications of polymers.

Polymer Name	Polymer Type	Key Properties	Specific Medical Applications
Poly(vinyl chloride) (PVC)	Synthetic	Versatile, easily sterilised	Tubing, blood bags, disposable devices [22]
Polypropylene (PP)	Synthetic	Durable, high-strength	Surgical trays, meshes, suture materials [23]
Ultra-High-Molecular-Weight Polyethylene (UHMWPE)	Synthetic	High strength-to-weight ratio, durability	Knee and hip replacement parts [24]
Polylactic Acid (PLA)	Synthetic	Biodegradable, biocompatible	Bone screws, sutures, vascular stents, and drug delivery [25]
Polyurethanes (PU)	Synthetic	Hemocompatible, tough, versatile	Catheters, wound dressings, artificial hearts, adhesives [26]
Polydopamine (PDA)	Natural-inspired	Biocompatible, adhesive, antimicrobial	Antimicrobial coatings, wound healing, and implant protection [27]
Poly(ether ether ketone) (PEEK)	Synthetic	High strength, radiolucent	Orthopaedic implants (bone screws, plates) [28]
Expanded PTFE (Gore-Tex^®^)	Synthetic	Chemically inert, porous structure	Vascular grafts, surgical meshes, ligament repair [29,30,31]
Chitosan	Natural	Biocompatible, biodegradable, antimicrobial	Wound healing, tissue engineering, and drug delivery [32]
Hyaluronic Acid (HA)	Natural	Biocompatible, mimics ECM	Wound healing, cartilage scaffolds, drug carriers [33,34]
Poly(ethylene glycol) (PEG)	Synthetic	Non-immunogenic, hydrophilic	Hydrogels, drug conjugates, coatings on devices [35,36,37]
Liquid Crystalline Polymers (LCPs)	Synthetic	High strength, lightweight, radiolucent	Minimally invasive surgical instruments, retinal implants [38,39]

**Table 3:** The most cited review articles on polymeric materials in biomedical engineering (>100 citations).

No.	Authors	Research Area	Main Focus	Key Findings	Times Cited
1	Zhang et al. [98]	Antimicrobial coatings; Biodegradable/biocompatible polymers; Drug delivery/Nanomedicine; Hydrogels/Biomaterials	Catechol-functionalized hydrogels for adhesion and biomedical use	Summarises recent advances and performance improvements. Highlights biocompatibility/safety considerations. Reports strong wet adhesion inspired by catechol/dopamine chemistry. Shows promise for wound healing and antimicrobial action. Identifies challenges and future research directions.	722
2	Nikolova & Chavali [99]	3D printing/Bioprinting; Tissue engineering/Regenerative medicine	Polymer-based scaffolds for tissue engineering/regeneration	Summarises recent advances and performance improvements. Notes applicability in 3D printing/bioprinting. Identifies challenges and future research directions.	709
3	Begines et al. [100]	Drug delivery/Nanomedicine; Oncology applications	Polymeric nanoparticles for controlled drug delivery	Summarises recent advances and performance improvements. Addresses oncology-oriented applications and efficacy. Identifies challenges and future research directions.	636
4	Reddy et al. [9]	Biodegradable/biocompatible polymers; Stimuli-responsive/Smart polymers; Tissue engineering/Regenerative medicine	Polymer-based scaffolds for tissue engineering/regeneration	Highlights biocompatibility/safety considerations.	626
5	Song et al. [1]	Biodegradable/biocompatible polymers; Drug delivery/Nanomedicine; Stimuli-responsive/Smart polymers; Tissue engineering/Regenerative medicine; Wound care/Antimicrobial	Polymer-based scaffolds for tissue engineering/regeneration	Summarises recent advances and performance improvements. Emphasises controlled/targeted drug release capabilities. Shows promise for wound healing and antimicrobial action.	590
6	Teo et al. [5]	Biodegradable/biocompatible polymers	Biomaterials polymer polymeric polymers	Highlights biocompatibility/safety considerations. Identifies challenges and future research directions.	581
7	Zhang et al. [101]	Biosensors/Diagnostics; Stimuli-responsive/Smart polymers	Polymer polymeric polymers	Summarises recent advances and performance improvements. Identifies challenges and future research directions.	533
8	Varaprasad et al. [102]	Hydrogels/Biomaterials; Stimuli-responsive/Smart polymers; Wound care/Antimicrobial	Polymer-based strategies for wound healing	Summarises recent advances and performance improvements. Shows promise for wound healing and antimicrobial action.	511
9	Islam et al. [103]	Biodegradable/biocompatible polymers; Stimuli-responsive/Smart polymers; Tissue engineering/Regenerative medicine	Polymer-based scaffolds for tissue engineering/regeneration	Summarises recent advances and performance improvements. Identifies challenges and future research directions.	467
10	Bai et al. [104]	Hydrogels/Biomaterials; Orthopedics; Stimuli-responsive/Smart polymers; Tissue engineering/Regenerative medicine; Wound care/Antimicrobial	Hydrogels for biomedical applications	Shows promise for wound healing and antimicrobial action. Identifies challenges and future research directions.	457
11	Kim & Matsunaga [105]	Hydrogels/Biomaterials; Stimuli-responsive/Smart polymers	Hydrogels for biomedical applications	Provides a consolidated overview of materials, methods and applications.	429
12	González-Henríquez [106]	3D printing/Bioprinting	3D printing/bioprinting of polymeric biomaterials	Notes applicability in 3D printing/bioprinting. Identifies challenges and future research directions.	383
13	Shaghaleh et al. [107]	Biomedical polymers/Biomaterials	Polymer polymeric polymers	Summarises recent advances and performance improvements. Identifies challenges and future research directions.	366
14	Karimi et al. [108]	Drug delivery/Nanomedicine; Stimuli-responsive/Smart polymers	Polymeric nanoparticles for controlled drug delivery	Emphasises controlled/targeted drug release capabilities.	344
15	Cho et al. [109]	Biodegradable/biocompatible polymers; Drug delivery/Nanomedicine; Hydrogels/Biomaterials; Stimuli-responsive/Smart polymers; Wound care/Antimicrobial	Hydrogels for biomedical applications	Highlights biocompatibility/safety considerations. Reports strong wet adhesion inspired by catechol/dopamine chemistry. Shows promise for wound healing and antimicrobial action.	308
16	Boni et al. [110]	Biodegradable/biocompatible polymers; Stimuli-responsive/Smart polymers; Tissue engineering/Regenerative medicine	Polymer-based scaffolds for tissue engineering/regeneration	Summarises recent advances and performance improvements. Highlights biocompatibility/safety considerations. Identifies challenges and future research directions.	292
17	Martins et al. [111]	Drug delivery/Nanomedicine; Stimuli-responsive/Smart polymers; Tissue engineering/Regenerative medicine	Polymer-based scaffolds for tissue engineering/regeneration	Provides a consolidated overview of materials, methods and applications.	291
18	Tang et al. [112]	Drug delivery/Nanomedicine; Tissue engineering/Regenerative medicine	Polymeric nanoparticles for controlled drug delivery	Summarises recent advances and performance improvements. Emphasises controlled/targeted drug release capabilities. Identifies challenges and future research directions.	288
19	Culmone et al. [113]	3D printing/Bioprinting; Biosensors/Diagnostics	3D printing/bioprinting of polymeric biomaterials	Notes applicability in 3D printing/bioprinting. Identifies challenges and future research directions.	285
20	Liao et al. [114]	Antimicrobial coatings; Biosensors/Diagnostics; Stimuli-responsive/Smart polymers	Biomaterials polymeric	Summarises recent advances and performance improvements. Highlights biocompatibility/safety considerations. Identifies challenges and future research directions.	264
21	Alghamdi [115]	3D printing/Bioprinting; Stimuli-responsive/Smart polymers	3D printing/bioprinting of polymeric biomaterials	Summarises recent advances and performance improvements. Notes applicability in 3D printing/bioprinting. Identifies challenges and future research directions.	262
22	Taylor et al. [116]	Tissue engineering/Regenerative medicine	Polymer-based scaffolds for tissue engineering/regeneration	Reports strong wet adhesion inspired by catechol/dopamine chemistry. Identifies challenges and future research directions.	241
23	Su et al. [117]	Biodegradable/biocompatible polymers; Drug delivery/Nanomedicine; Stimuli-responsive/Smart polymers; Tissue engineering/Regenerative medicine	Polymer-based scaffolds for tissue engineering/regeneration	Highlights biocompatibility/safety considerations. Emphasises controlled/targeted drug release capabilities. Identifies challenges and future research directions.	221
24	Bagheri et al. [118]	Dental; Drug delivery/Nanomedicine; Stimuli-responsive/Smart polymers	Polymeric nanoparticles for controlled drug delivery	Summarises recent advances and performance improvements. Emphasises controlled/targeted drug release capabilities. Identifies challenges and future research directions.	220
25	Bernard et al. [119]	Biodegradable/biocompatible polymers; Drug delivery/Nanomedicine; Stimuli-responsive/Smart polymers	Controlled drug delivery with polymer platforms	Highlights biocompatibility/safety considerations. Reports strong wet adhesion inspired by catechol/dopamine chemistry. Emphasises controlled/targeted drug release capabilities. Identifies challenges and future research directions.	215
26	Wo et al. [120]	Antimicrobial coatings; Stimuli-responsive/Smart polymers	Delivery polymer polymeric polymers	Summarises recent advances and performance improvements. Reports strong wet adhesion inspired by catechol/dopamine chemistry. Emphasises controlled/targeted drug release capabilities.	213
27	Beg et al. [121]	Biodegradable/biocompatible polymers; Drug delivery/Nanomedicine; Stimuli-responsive/Smart polymers	Biodegradable polymer systems for medical use	Provides a consolidated overview of materials, methods and applications.	212
28	Wells et al. [122]	Cardiovascular; Dental; Stimuli-responsive/Smart polymers	Stimuli-responsive/smart polymer systems	Summarises recent advances and performance improvements. Emphasises controlled/targeted drug release capabilities.	203
29	Sánchez-Cid et al. [123]	Biodegradable/biocompatible polymers; Drug delivery/Nanomedicine; Hydrogels/Biomaterials; Tissue engineering/Regenerative medicine; Wound care/Antimicrobial	Hydrogels for biomedical applications	Summarises recent advances and performance improvements. Highlights biocompatibility/safety considerations. Emphasises controlled/targeted drug release capabilities. Shows promise for wound healing and antimicrobial action. Identifies challenges and future research directions.	198
30	Mushtaq et al. [124]	Antimicrobial coatings; Biosensors/Diagnostics; Drug delivery/Nanomedicine; Hydrogels/Biomaterials; Stimuli-responsive/Smart polymers; Tissue engineering/Regenerative medicine; Wound care/Antimicrobial	Hydrogels for biomedical applications	Summarises recent advances and performance improvements. Emphasises controlled/targeted drug release capabilities. Notes applicability in 3D printing/bioprinting. Shows promise for wound healing and antimicrobial action. Identifies challenges and future research directions.	193
31	Zhang et al. [125]	3D printing/Bioprinting; Biosensors/Diagnostics; Stimuli-responsive/Smart polymers	Polymeric	Summarises recent advances and performance improvements. Notes applicability in 3D printing/bioprinting. Identifies challenges and future research directions.	193
32	Marco-Dufort & Tibbitt [126]	Hydrogels/Biomaterials; Orthopedics; Stimuli-responsive/Smart polymers; Tissue engineering/Regenerative medicine	Hydrogels for biomedical applications	Provides a consolidated overview of materials, methods and applications.	177
33	Wang et al. [127]	Stimuli-responsive/Smart polymers	Stimuli-responsive/smart polymer systems	Summarises recent advances and performance improvements.	172
34	Kenry Liu [128]	Biodegradable/biocompatible polymers; Tissue engineering/Regenerative medicine	Biodegradable polymer systems for medical use	Summarises recent advances and performance improvements. Highlights biocompatibility/safety considerations. Identifies challenges and future research directions.	169
35	Khan et al. [129]	Drug delivery/Nanomedicine; Oncology applications; Stimuli-responsive/Smart polymers	Polymeric nanoparticles for controlled drug delivery	Summarises recent advances and performance improvements. Emphasises controlled/targeted drug release capabilities. Addresses oncology-oriented applications and efficacy. Identifies challenges and future research directions.	166
36	Essa et al. [130]	Biodegradable/biocompatible polymers; Drug delivery/Nanomedicine	Controlled drug delivery with polymer platforms	Highlights biocompatibility/safety considerations.	164
37	Kennedy et al. [131]	Biodegradable/biocompatible polymers; Stimuli-responsive/Smart polymers; Tissue engineering/Regenerative medicine	Polymer-based scaffolds for tissue engineering/regeneration	Summarises recent advances and performance improvements. Identifies challenges and future research directions.	164
38	Long et al. [132]	Stimuli-responsive/Smart polymers; Tissue engineering/Regenerative medicine	Polymeric biomaterials overview	Identifies challenges and future research directions.	164
39	Tipnis & Burgess [133]	Biodegradable/biocompatible polymers	Biodegradable polymer systems for medical use	Highlights biocompatibility/safety considerations.	162
40	Abbasian et al. [134]	Biodegradable/biocompatible polymers; Stimuli-responsive/Smart polymers; Tissue engineering/Regenerative medicine	Polymer-based scaffolds for tissue engineering/regeneration	Summarises recent advances and performance improvements. Highlights biocompatibility/safety considerations. Identifies challenges and future research directions.	160
41	Behera & Mahanwar [135]	Biodegradable/biocompatible polymers; Biosensors/Diagnostics; Drug delivery/Nanomedicine; Hydrogels/Biomaterials; Stimuli-responsive/Smart polymers; Tissue engineering/Regenerative medicine	Hydrogels for biomedical applications	Highlights biocompatibility/safety considerations. Emphasises controlled/targeted drug release capabilities.	159
42	Sharma et al. [136]	Antimicrobial coatings; Biodegradable/biocompatible polymers; Dental; Drug delivery/Nanomedicine; Oncology applications; Stimuli-responsive/Smart polymers; Tissue engineering/Regenerative medicine; Wound care/Antimicrobial	Polymer-based scaffolds for tissue engineering/regeneration	Summarises recent advances and performance improvements. Highlights biocompatibility/safety considerations. Emphasises controlled/targeted drug release capabilities. Shows promise for wound healing and antimicrobial action. Addresses oncology-oriented applications and efficacy.	157
43	Tang et al. [137]	Oncology applications; Stimuli-responsive/Smart polymers; Wound care/Antimicrobial	Stimuli-responsive/smart polymer systems	Summarises recent advances and performance improvements. Emphasises controlled/targeted drug release capabilities. Addresses oncology-oriented applications and efficacy.	157
44	Kumar et al. [138]	Antimicrobial coatings; Biodegradable/biocompatible polymers; Drug delivery/Nanomedicine; Tissue engineering/Regenerative medicine; Wound care/Antimicrobial	Biodegradable polymer systems for medical use	Summarises recent advances and performance improvements. Highlights biocompatibility/safety considerations. Shows promise for wound healing and antimicrobial action.	155
45	Moussa & Aparicio [139]	Antimicrobial coatings; Dental; Stimuli-responsive/Smart polymers; Tissue engineering/Regenerative medicine	Polymer-based scaffolds for tissue engineering/regeneration	Summarises recent advances and performance improvements. Identifies challenges and future research directions.	152
46	Schneider et al. [140]	Biodegradable/biocompatible polymers; Drug delivery/Nanomedicine	Controlled drug delivery with polymer platforms	Highlights biocompatibility/safety considerations. Emphasises controlled/targeted drug release capabilities.	149
47	Eivazzadeh-Keihan et al. [141]	Orthopedics; Stimuli-responsive/Smart polymers; Tissue engineering/Regenerative medicine	Polymer-based scaffolds for tissue engineering/regeneration	Highlights biocompatibility/safety considerations.	149
48	Esrafili et al. [142]	Drug delivery/Nanomedicine	Controlled drug delivery with polymer platforms	Provides a consolidated overview of materials, methods and applications.	148
49	Zhang [143]	Stimuli-responsive/Smart polymers	Polymeric polymerization; polymer replacement	Summarises recent advances and performance improvements. Identifies challenges and future research directions.	148
50	Nouri et al. [144]	3D printing/Bioprinting; Dental; Orthopedics; Tissue engineering/Regenerative medicine	Polymer-based scaffolds for tissue engineering/regeneration	Notes applicability in 3D printing/bioprinting.	146
51	Chen et al. [145]	Oncology applications; Tissue engineering/Regenerative medicine	Biomaterials delivery polymers	Summarises recent advances and performance improvements. Addresses oncology-oriented applications and efficacy. Identifies challenges and future research directions.	144
52	Dias et al. [146]	Stimuli-responsive/Smart polymers; Tissue engineering/Regenerative medicine; Wound care/Antimicrobial	Polymer-based strategies for wound healing	Summarises recent advances and performance improvements. Shows promise for wound healing and antimicrobial action. Identifies challenges and future research directions.	144
53	Qadir et al. [147]	Biodegradable/biocompatible polymers; Dental; Orthopedics; Stimuli-responsive/Smart polymers; Tissue engineering/Regenerative medicine	Biodegradable polymer systems for medical use	Summarises recent advances and performance improvements. Highlights biocompatibility/safety considerations. Reports strong wet adhesion inspired by catechol/dopamine chemistry. Identifies challenges and future research directions.	144
54	Dziadek et al. [148]	Biodegradable/biocompatible polymers; Stimuli-responsive/Smart polymers	Biodegradable polymer systems for medical use	Provides a consolidated overview of materials, methods and applications.	143
55	Mann et al. [149]	3D printing/Bioprinting; Drug delivery/Nanomedicine; Stimuli-responsive/Smart polymers; Tissue engineering/Regenerative medicine; Wound care/Antimicrobial	Stimuli-responsive/smart polymer systems	Summarises recent advances and performance improvements. Emphasises controlled/targeted drug release capabilities. Notes applicability in 3D printing/bioprinting. Shows promise for wound healing and antimicrobial action.	142
56	Arif et al. [150]	3D printing/Bioprinting; Biodegradable/biocompatible polymers; Drug delivery/Nanomedicine; Stimuli-responsive/Smart polymers; Tissue engineering/Regenerative medicine; Wound care/Antimicrobial	Polymeric nanoparticles for controlled drug delivery	Summarises recent advances and performance improvements. Highlights biocompatibility/safety considerations. Emphasises controlled/targeted drug release capabilities. Notes applicability in 3D printing/bioprinting. Shows promise for wound healing and antimicrobial action. Identifies challenges and future research directions.	140
57	Peng et al. [151]	Biodegradable/biocompatible polymers; Orthopedics; Stimuli-responsive/Smart polymers; Tissue engineering/Regenerative medicine	Polymer-based scaffolds for tissue engineering/regeneration	Summarises recent advances and performance improvements. Highlights biocompatibility/safety considerations. Identifies challenges and future research directions.	140
58	Rokaya et al. [152]	Antimicrobial coatings; Dental; Drug delivery/Nanomedicine; Tissue engineering/Regenerative medicine	Controlled drug delivery with polymer platforms	Summarises recent advances and performance improvements. Reports strong wet adhesion inspired by catechol/dopamine chemistry. Shows promise for wound healing and antimicrobial action. Identifies challenges and future research directions.	138
59	Elmowafy et al. [153]	Drug delivery/Nanomedicine; Oncology applications; Stimuli-responsive/Smart polymers	Polymeric nanoparticles for controlled drug delivery	Summarises recent advances and performance improvements. Emphasises controlled/targeted drug release capabilities. Addresses oncology-oriented applications and efficacy. Identifies challenges and future research directions.	137
60	Cai et al. [154]	3D printing/Bioprinting; Antimicrobial coatings; Biodegradable/biocompatible polymers; Hydrogels/Biomaterials; Stimuli-responsive/Smart polymers; Tissue engineering/Regenerative medicine; Wound care/Antimicrobial	Hydrogels for biomedical applications	Summarises recent advances and performance improvements. Reports strong wet adhesion inspired by catechol/dopamine chemistry. Notes applicability in 3D printing/bioprinting. Shows promise for wound healing and antimicrobial action. Identifies challenges and future research directions.	136
61	Kaniuk & Stachewicz [155]	Biodegradable/biocompatible polymers; Drug delivery/Nanomedicine; Stimuli-responsive/Smart polymers; Tissue engineering/Regenerative medicine; Wound care/Antimicrobial	Polymer-based scaffolds for tissue engineering/regeneration	Summarises recent advances and performance improvements. Highlights biocompatibility/safety considerations. Notes applicability in 3D printing/bioprinting. Shows promise for wound healing and antimicrobial action.	134
62	Bonakdar & Rodrigue [156]	Stimuli-responsive/Smart polymers	Polymeric	Summarises recent advances and performance improvements.	134
63	Cook & Perrier [157]	Drug delivery/Nanomedicine	Controlled drug delivery with polymer platforms	Summarises recent advances and performance improvements.	133
64	Sionkowska et al. [158]	Biomedical polymers/Biomaterials	Polymeric biomaterials overview	Summarises recent advances and performance improvements.	132
65	Rother et al. [159]	Drug delivery/Nanomedicine; Stimuli-responsive/Smart polymers	Polymeric nanoparticles for controlled drug delivery	Emphasises controlled/targeted drug release capabilities. Identifies challenges and future research directions.	132
66	Gnanasekar [160]	Antimicrobial coatings; Hydrogels/Biomaterials; Stimuli-responsive/Smart polymers; Tissue engineering/Regenerative medicine	Hydrogels for biomedical applications	Summarises recent advances and performance improvements. Identifies challenges and future research directions.	132
67	Naikwadi et al. [161]	Orthopedics; Stimuli-responsive/Smart polymers; Tissue engineering/Regenerative medicine	Polymer polymeric polymers	Summarises recent advances and performance improvements. Emphasises controlled/targeted drug release capabilities.	131
68	Amiri et al. [162]	Drug delivery/Nanomedicine; Stimuli-responsive/Smart polymers	Controlled drug delivery with polymer platforms	Summarises recent advances and performance improvements. Highlights biocompatibility/safety considerations. Reports strong wet adhesion inspired by catechol/dopamine chemistry.	131
69	Ortega et al. [163]	Biodegradable/biocompatible polymers; Drug delivery/Nanomedicine; Tissue engineering/Regenerative medicine	Polymeric nanoparticles for controlled drug delivery	Provides a consolidated overview of materials, methods and applications.	131
70	Arif et al. [164]	3D printing/Bioprinting; Drug delivery/Nanomedicine; Hydrogels/Biomaterials; Orthopedics; Stimuli-responsive/Smart polymers; Tissue engineering/Regenerative medicine	Hydrogels for biomedical applications	Notes applicability in 3D printing/bioprinting. Identifies challenges and future research directions.	129
71	Rong et al. [165]	Antimicrobial coatings; Hydrogels/Biomaterials; Stimuli-responsive/Smart polymers	Hydrogels for biomedical applications	Emphasises controlled/targeted drug release capabilities. Shows promise for wound healing and antimicrobial action. Identifies challenges and future research directions.	127
72	Su et al. [166]	Biodegradable/biocompatible polymers	Biomaterial biomaterials polymer polymeric	Highlights biocompatibility/safety considerations. Identifies challenges and future research directions.	123
73	Tan [167]	Wound care/Antimicrobial	Polymeric biomaterials overview	Summarises recent advances and performance improvements. Shows promise for wound healing and antimicrobial action. Identifies challenges and future research directions.	122
74	Zhang et al. [168]	Drug delivery/Nanomedicine; Oncology applications; Stimuli-responsive/Smart polymers	Controlled drug delivery with polymer platforms	Summarises recent advances and performance improvements. Addresses oncology-oriented applications and efficacy.	121
75	Scognamiglio [169]	Hydrogels/Biomaterials; Stimuli-responsive/Smart polymers; Wound care/Antimicrobial	Polymer-based strategies for wound healing	Reports strong wet adhesion inspired by catechol/dopamine chemistry. Emphasises controlled/targeted drug release capabilities. Shows promise for wound healing and antimicrobial action. Identifies challenges and future research directions.	121
76	Shrimal et al. [170]	Drug delivery/Nanomedicine	Polymeric nanoparticles for controlled drug delivery	Summarises recent advances and performance improvements. Emphasises controlled/targeted drug release capabilities. Identifies challenges and future research directions.	120
77	Kim & Meng [171]	Biomedical polymers/Biomaterials	Polymer polymeric polymers	Summarises recent advances and performance improvements. Emphasises controlled/targeted drug release capabilities.	119
78	Asa’ad et al. [172]	Dental; Orthopaedics; Tissue engineering/Regenerative medicine	Polymer-based scaffolds for tissue engineering/regeneration	Identifies challenges and future research directions.	118
79	Shcherbakov et al. [173]	Biodegradable/biocompatible polymers; Drug delivery/Nanomedicine; Hydrogels/Biomaterials	Hydrogels for biomedical applications	Summarises recent advances and performance improvements.	117
80	Venkatesan et al. [174]	Biodegradable/biocompatible polymers; Drug delivery/Nanomedicine; Hydrogels/Biomaterials; Oncology applications	Polymeric nanoparticles for controlled drug delivery	Summarises recent advances and performance improvements. Highlights biocompatibility/safety considerations. Emphasises controlled/targeted drug release capabilities. Addresses oncology-oriented applications and efficacy.	117
81	Alvarez-Paino et al. [175]	Antimicrobial coatings	Polymer polymeric polymers	Shows promise for wound healing and antimicrobial action.	117
82	Patil & Kandasubramanian [176]	Antimicrobial coatings; Biodegradable/biocompatible polymers; Drug delivery/Nanomedicine; Hydrogels/Biomaterials; Oncology applications; Stimuli-responsive/Smart polymers; Wound care/Antimicrobial	Hydrogels for biomedical applications	Summarises recent advances and performance improvements. Highlights biocompatibility/safety considerations. Reports strong wet adhesion inspired by catechol/dopamine chemistry. Emphasises controlled/targeted drug release capabilities. Shows promise for wound healing and antimicrobial action. Addresses oncology-oriented applications and efficacy.	111
83	Cross et al. [177]	Orthopedics; Stimuli-responsive/Smart polymers; Tissue engineering/Regenerative medicine	Biomaterials polymeric	Summarises recent advances and performance improvements. Identifies challenges and future research directions.	111
84	Teleky & Vodnar [178]	Biodegradable/biocompatible polymers; Drug delivery/Nanomedicine; Hydrogels/Biomaterials; Oncology applications; Stimuli-responsive/Smart polymers	Hydrogels for biomedical applications	Emphasises controlled/targeted drug release capabilities. Addresses oncology-oriented applications and efficacy.	109
85	Olmos & González-Benito [179]	Antimicrobial coatings; Stimuli-responsive/Smart polymers	Polymer polymeric	Provides a consolidated overview of materials, methods and applications.	108
86	Mohamadhoseini & Mohamadnia [180]	Biodegradable/biocompatible polymers; Drug delivery/Nanomedicine; Stimuli-responsive/Smart polymers; Wound care/Antimicrobial	Stimuli-responsive/smart polymer systems	Highlights biocompatibility/safety considerations. Shows promise for wound healing and antimicrobial action. Identifies challenges and future research directions.	107
87	Arif et al. [181]	Hydrogels/Biomaterials; Stimuli-responsive/Smart polymers; Tissue engineering/Regenerative medicine; Wound care/Antimicrobial	Polymer-based strategies for wound healing	Summarises recent advances and performance improvements. Shows promise for wound healing and antimicrobial action. Identifies challenges and future research directions.	106
88	Chahal et al. [182]	Biodegradable/biocompatible polymers; Orthopedics; Tissue engineering/Regenerative medicine	Polymer-based scaffolds for tissue engineering/regeneration	Highlights biocompatibility/safety considerations. Identifies challenges and future research directions.	104
89	Hussain [183]	Antimicrobial coatings; Cardiovascular; Drug delivery/Nanomedicine; Hydrogels/Biomaterials; Stimuli-responsive/Smart polymers; Tissue engineering/Regenerative medicine; Wound care/Antimicrobial	Hydrogels for biomedical applications	Summarises recent advances and performance improvements. Shows promise for wound healing and antimicrobial action. Identifies challenges and future research directions.	102
90	Rahimi [184]	Drug delivery/Nanomedicine; Hydrogels/Biomaterials; Orthopedics; Tissue engineering/Regenerative medicine	Hydrogels for biomedical applications	Summarises recent advances and performance improvements. Emphasises controlled/targeted drug release capabilities. Identifies challenges and future research directions.	101

**Table 4:** Progress, challenges, and future directions in polymeric biomaterials.

Aspect	Key Points	References
Progress	Substantial advances are evident in scaffold design for tissue engineering, particularly in ECM-mimetic hydrogels, composites, and electrospun architectures that support cell adhesion, proliferation, and differentiation.	[9,99,103,104,110,131]
	In drug delivery and nanomedicine, polymeric nanoparticles and stimuli-responsive systems have enabled controlled release and on-demand activation, with strong momentum in oncology and chronic disease applications.	[100,108,112,118,129,130]
	Wound healing and antimicrobial strategies have advanced through the use of catechol-functionalized hydrogels, chitosan-based composites, and nitric oxide–releasing platforms that integrate tissue repair with infection control.	[98,99,109,123,136,154]
	Additive manufacturing—encompassing 3D/4D printing and bioprinting—has expanded the design space to include patient-specific implants and complex, multifunctional scaffolds.	[106,113,144,150,166].
Challenges	Long-term biocompatibility and safety, together with clear regulatory pathways, remain central hurdles for clinical translation.	[119]
	Mechanical integration and durability at the cell–matrix interface remain challenging to guarantee across various tissues and loading conditions, while trade-offs between bioactivity and reproducibility persist for constructs based on natural macromolecules.	[131,134]
	Scale-up and batch-to-batch reproducibility for advanced polymer systems (including PLGA carriers, COF-based platforms, and phytochemical-loaded nanoparticles) are not yet resolved.	[100,130,142,153]
	Comparative clinical performance and cost-effectiveness versus standard of care are insufficiently documented, particularly for wound-care materials and complex hydrogel systems.	[102,123,136]
Future directions	The literature converges on hybrid platforms that integrate natural and synthetic components to marry bioactivity with mechanical/processing robustness.	[110,134]
	Broader adoption of patient-specific bioprinting and 4D-printed constructs to tailor form, function, and degradation in vivo.	[106,113,144,150,164]
	Continued refinement of stimuli-responsive and multifunctional nanomedicine platforms to improve spatiotemporal control and therapeutic indices.	[108,112,118,142]

## Data Availability

The original contributions presented in this study are included in the article. Further inquiries can be directed to the corresponding author.

## Supplementary Materials

The following supporting information can be downloaded at: https://www.mdpi.com/article/10.3390/polym17212886/s1, Table S1: PRISMA 2020 Checklist.
