# Supplementary material for: Polymeric Materials in Biomedical Engineering: A Bibliometric Mapping
Source: Polymers (Basel). 2025 Oct 29;17(21):2886. doi: 10.3390/polym17212886 (PMC12608308; doi:10.3390/polym17212886)
Supplement: Supplementary file 1 [file polymers-17-02886-s001.zip › polymers-3929053-supplementary.pdf]

PRISMA 2020 Checklist

Table S1.PRISMA 2020 Checklist

Section and Topic	Item #	Checklist item	Location where item is reported
**TITLE**			
Title	1	Identify the report as a systematic review.	Polymeric Materials in Biomedical Engineering: A Bibliometric Mapping and Review of Reviews.
**ABSTRACT**			
Abstract	2	See the PRISMA 2020 for Abstracts checklist.	Presents background, methods, results, and conclusions.
**INTRODUCTION**			
Rationale	3	Describe the rationale for the review in the context of existing knowledge.	Contextualises evolution of polymeric biomaterials and scientific need for synthesis.
Objectives	4	Provide an explicit statement of the objective(s) or question(s) the review addresses.	To identify key findings and knowledge gaps.
**METHODS**			
Eligibility criteria	5	Specify the inclusion and exclusion criteria for the review and how studies were grouped for the syntheses.	“Materials and Methods” – specifies inclusion (reviews 2016–2025, biomedical focus) and exclusion (non-biomedical domains) criteria; Figure 1 PRISMA flow diagram.
Information sources	6	Specify all databases, registers, websites, organisations, reference lists and other sources searched or consulted to identify studies. Specify the date when each source was last searched or consulted.	Web of Science Core Collection, date range 2016–2025.
Search strategy	7	Present the full search strategies for all databases, registers and websites, including any filters and limits used.	The search was conducted using the query ("polymeric materials" OR "biomedical polymers" OR "polymeric biomaterials") AND ("biomedical" OR "medical" OR "healthcare" OR "tissue engineering" OR "drug delivery" OR "implant") AND (Review OR "Systematic Review")
Selection process	8	Specify the methods used to decide whether a study met the inclusion criteria of the review, including how many reviewers screened each record and each report retrieved, whether they worked independently, and if applicable, details of automation tools used in the process.	Section 3, paragraphs 4–6 and Figure 1. The authors manually screened all 1,095 records. Non-relevant studies were excluded after title/abstract screening and full-text reading. Manual validation confirmed final inclusion of 589 review articles. No automation tools or multiple independent reviewers were involved; screening was performed by the authors collaboratively.

Section and Topic	Item #	Checklist item	Location where item is reported
Data collection process	9	Specify the methods used to collect data from reports, including how many reviewers collected data from each report, whether they worked independently, any processes for obtaining or confirming data from study investigators, and if applicable, details of automation tools used in the process.	Section 3, last paragraph. Data were extracted manually from the bibliographic metadata (authors, titles, year, citation count, research area, and key findings). The process included manual verification of the top 90 most cited reviews (≥100 citations). Data for bibliometric mapping were imported into *VOSviewer* for network analysis.
Data items	10a	List and define all outcomes for which data were sought. Specify whether all results that were compatible with each outcome domain in each study were sought (e.g. for all measures, time points, analyses), and if not, the methods used to decide which results to collect.	Sections 3–4. The outcomes analyzed included bibliometric indicators (keyword co-occurrence, bibliographic coupling, international collaboration networks) and qualitative synthesis outcomes (major research themes and challenges). Figures 3–8 show these visual analyses.
	10b	List and define all other variables for which data were sought (e.g. participant and intervention characteristics, funding sources). Describe any assumptions made about any missing or unclear information.	Section 4 (“Bibliometric Analysis”). Variables included journal sources, average publication year, country-level collaboration, and thematic clustering. No participant- or intervention-level variables exist because this was not a clinical review.
Study risk of bias assessment	11	Specify the methods used to assess risk of bias in the included studies, including details of the tool(s) used, how many reviewers assessed each study and whether they worked independently, and if applicable, details of automation tools used in the process.	Not applicable. The study used bibliometric and narrative synthesis of review papers, not primary research; no bias assessment tool (e.g., ROBIS) was applied.
Effect measures	12	Specify for each outcome the effect measure(s) (e.g. risk ratio, mean difference) used in the synthesis or presentation of results.	Not applicable. No quantitative synthesis or meta-analysis was performed; results are descriptive and thematic.
Synthesis methods	13a	Describe the processes used to decide which studies were eligible for each synthesis (e.g. tabulating the study intervention characteristics and comparing against the planned groups for each synthesis (item #5)).	Section 3. The synthesis included all *review* papers matching inclusion criteria, with additional in-depth qualitative synthesis of the *90 most cited reviews* (≥100 citations) as representative of the field’s key directions.
	13b	Describe any methods required to prepare the data for presentation or synthesis, such as handling of missing summary statistics, or data conversions.	Section 3. Bibliometric data were standardized and imported into *VOSviewer* (version 1.6.20) for co-occurrence and coupling analyses.

Section and Topic	Item #	Checklist item	Location where item is reported
			No imputation or conversion of numerical data was required.
	13c	Describe any methods used to tabulate or visually display results of individual studies and syntheses.	Figures 2–8 and Tables 3–4. Results are presented as bibliometric maps (co-occurrence, overlay, density, and co-authorship) and summary tables for the 90 top-cited reviews, detailing focus area, key findings, and citations.
	13d	Describe any methods used to synthesize results and provide a rationale for the choice(s). If meta-analysis was performed, describe the model(s), method(s) to identify the presence and extent of statistical heterogeneity, and software package(s) used.	Sections 3–5. A *mixed bibliometric–narrative* approach was applied:<br>1. Quantitative mapping using VOSviewer to visualize research structures.<br>2. Qualitative synthesis summarizing dominant themes and methodological trends.<br>No statistical meta-analysis was conducted.
	13e	Describe any methods used to explore possible causes of heterogeneity among study results (e.g. subgroup analysis, meta-regression).	Not applicable. Heterogeneity was conceptual rather than statistical; diversity in thematic clusters was described in Section 4.
	13f	Describe any sensitivity analyses conducted to assess robustness of the synthesized results.	Not applicable. No statistical sensitivity analyses were relevant to this study design.
Reporting bias assessment	14	Describe any methods used to assess risk of bias due to missing results in a synthesis (arising from reporting biases).	Not applicable. The review analyzed bibliometric data; no missing-results or publication bias analysis was feasible.
Certainty assessment	15	Describe any methods used to assess certainty (or confidence) in the body of evidence for an outcome.	Not applicable. Certainty or confidence assessment (e.g., GRADE) is not applicable to bibliometric or review-of-reviews designs.
**RESULTS**			
Study selection	16a	Describe the results of the search and selection process, from the number of records identified in the search to the number of studies included in the review, ideally using a flow diagram.	Section 3 and Figure 1 (PRISMA flow diagram). Out of 1,095 records, 467 were excluded, 628 full-texts assessed, and 589 review articles finally included. The process is fully documented in Figure 1.
	16b	Cite studies that might appear to meet the inclusion criteria, but which were excluded, and explain why they were excluded.	Section 3. Explicitly enumerates excluded categories: chemistry (15), energy/environment (7), plant/marine

Section and Topic	Item #	Checklist item	Location where item is reported
			(3), nanomaterials (5), non-biomedical engineering (4), electronics (3), and miscellaneous (2).
Study characteristics	17	Cite each included study and present its characteristics.	Table 3 (pp. ~20–30). Each of the 90 most cited reviews is listed with author(s), area, focus, key findings, and citation count.
Risk of bias in studies	18	Present assessments of risk of bias for each included study.	Not applicable. Review of reviews without primary data; no bias scoring conducted.
Results of individual studies	19	For all outcomes, present, for each study: (a) summary statistics for each group (where appropriate) and (b) an effect estimate and its precision (e.g. confidence/credible interval), ideally using structured tables or plots.	Table 3 – summarizes each included review's thematic focus and findings.
Results of syntheses	20a	For each synthesis, briefly summarise the characteristics and risk of bias among contributing studies.	Sections 4–5. Summarizes bibliometric mapping (keyword clusters, journal coupling, co-authorship) and thematic synthesis of review findings.
	20b	Present results of all statistical syntheses conducted. If meta-analysis was done, present for each the summary estimate and its precision (e.g. confidence/credible interval) and measures of statistical heterogeneity. If comparing groups, describe the direction of the effect.	Not applicable – no meta-analysis or effect size aggregation.
	20c	Present results of all investigations of possible causes of heterogeneity among study results.	Section 4 – thematic variability illustrated via color-coded clusters in VOSviewer maps (Figures 3–6).
	20d	Present results of all sensitivity analyses conducted to assess the robustness of the synthesized results.	Not applicable – descriptive mapping only.
Reporting biases	21	Present assessments of risk of bias due to missing results (arising from reporting biases) for each synthesis assessed.	Not applicable – bibliometric data are complete within the Web of Science Core Collection.
Certainty of evidence	22	Present assessments of certainty (or confidence) in the body of evidence for each outcome assessed.	Not applicable – descriptive and bibliometric results only.
**DISCUSSION**			
Discussion	23a	Provide a general interpretation of the results in the context of other evidence.	Section 6 ("Conclusions") – integrates bibliometric and narrative findings, summarizing the four major research domains (tissue engineering, drug delivery, wound healing, additive manufacturing).
	23b	Discuss any limitations of the evidence included in the review.	Section 6 – explicitly acknowledges limitations of dataset (restricted to Web of Science, potential omission of Scopus/PubMed records, and focus on reviews only).
	23c	Discuss any limitations of the review processes used.	Section 6 – discusses that

PRISMA 2020 Checklist

Section and Topic	Item #	Checklist item	Location where item is reported
			inclusion/exclusion was manual and that the analysis reflects trends rather than exhaustive coverage.
	23d	Discuss implications of the results for practice, policy, and future research.	Section 6 – final paragraphs emphasize interdisciplinary integration, regulatory harmonization, and translational opportunities for biomedical polymers.
**OTHER INFORMATION**			
Registration and protocol	24a	Provide registration information for the review, including register name and registration number, or state that the review was not registered.	
	24b	Indicate where the review protocol can be accessed, or state that a protocol was not prepared.	
	24c	Describe and explain any amendments to information provided at registration or in the protocol.	
Support	25	Describe sources of financial or non-financial support for the review, and the role of the funders or sponsors in the review.	Acknowledgment section (end of manuscript): *"The authors received no external funding."* (or specify if later added).
Competing interests	26	Declare any competing interests of review authors.	The authors declare no conflict of interest.
Availability of data, code and other materials	27	Report which of the following are publicly available and where they can be found: template data collection forms; data extracted from included studies; data used for all analyses; analytic code; any other materials used in the review.	Data Availability Statement (end of article): *"All bibliometric data were retrieved from the Web of Science Core Collection and are available from the corresponding author upon request".*

From: Page MJ, McKenzie JE, Bossuyt PM, Boutron I, Hoffmann TC, Mulrow CD, et al. The PRISMA 2020 statement: an updated guideline for reporting systematic reviews. BMJ 2021;372:n71. doi: 10.1136/bmj.n71. This work is licensed under CC BY 4.0. To view a copy of this license, visit https://creativecommons.org/licenses/by/4.0/
